# Mapping the distribution of health equity research and practice across a university: a network analysis

**DOI:** 10.1017/cts.2023.555

**Published:** 2023-05-22

**Authors:** Reza Yousefi Nooraie, Porooshat Dadgostar, Gretchen Roman, John P. Cullen, Nancy M. Bennett

**Affiliations:** 1 Department of Public Health Sciences, University of Rochester, Rochester, NY, USA; 2 Clinical and Translational Science Institute, University of Rochester School of Medicine and Dentistry, Rochester, NY, USA; 3 Department of Family Medicine, University of Rochester, Rochester, NY, USA; 4 Susan B. Anthony Center, University of Rochester, Rochester, NY, USA; 5 Center for Community Health and Prevention, University of Rochester Medical Center, Rochester, NY, USA

**Keywords:** Social network analysis, health equity, anti-racism, translational science, invisible communities

## Abstract

**Introduction:**

Health equity research spans various disciplines, crossing formal organizational and departmental barriers and forming invisible communities. This study aimed to map the nomination network of scholars at the University of Rochester Medical Center who were active in racial and ethnic health equity research, education, and social/administrative activities, to identify the predictors of peer recognition.

**Methods:**

We conducted a snowball survey of faculty members with experience and/or interest in racial and ethnic health equity, nominating peers with relevant expertise.

**Results:**

Data from a total of 121 individuals (64% doing research on extent and outcomes of racial/ethnic disparities and racism, 48% research on interventions, 55% education, and 50% social/administrative activities) were gathered in six rounds of survey. The overlap between expertise categories was small with coincidence observed between education and social/administrative activities (kappa: 0.27; *p* < 0.001). Respondents were more likely to nominate someone if both were involved in research (OR: 3.1), if both were involved in education (OR: 1.7), and if both were affiliated with the same department (OR: 3.7). Being involved in health equity research significantly predicted the centrality of an individual in the nomination network, and the most central actors were involved in multiple expertise categories.

**Conclusions:**

Compared with equity researchers, those involved in racial equity social/administrative activities were less likely to be recognized by peers as equity experts.

## Introduction

Achieving health equity, or fair opportunities for the best possible health for all members of society regardless of their race, ethnicity, and other social determinants, has been a priority for policy and research [1,2]. Given the persisting trends of health inequities in the USA [3,4], different health and social science research fields, from basic sciences to epidemiology and public policy, have raised the call for increased attention to and awareness of health equity research through an antiracist lens [5–7].

There are still substantial gaps in our knowledge regarding the complexity of interrelated and multifaceted social, health, and systemic determinants of health equity, and effective actions to achieve it [8–11]. Health equity research is multidisciplinary and translational, as it recognizes the multilevel and embedded nature of context and unique needs and characteristics of populations [12]. To effectively tackle this complexity, translational, and transdisciplinary research is needed to meaningfully engage and activate various partners [13–17]. Researchers from various disciplines, administrators, and implementers of interventions and policies, and community members facing health inequities should be engaged to make sure the questions, data, methods, and findings are relevant and useful to real-world settings [13]. As a result, the majority of the Clinical and Translational Science Awards (CTSA) consortium leaders identified health equity research as a priority and stated their commitment to facilitate more health equity research supported by the CTSA programs [18].

Expertise and interest in health equity, similar to other translational concepts, is distributed across various basic sciences, clinical, and population health disciplines, crossing formal organizational and departmental barriers and forming invisible communities [19]. Invisible communities (also known as “invisible colleges” and “epistemic communities”) [20] are comprised of researchers who collaborate and cooperate based on their mutual interests [19] and may go beyond traditional institutional boundaries. These communities play an essential role in the production of knowledge and generation of innovations [21]. Network analysis offers a novel methodology for understanding the structure and configuration of these invisible communities [22,23]. By identifying the invisible communities and understanding their compositions and dynamics, academic institutions can develop tailored interventions to empower and bridge communities of expertise and facilitate knowledge sharing and dialog, towards building capacity for translational and cross-disciplinary innovations.

We aimed to understand the distribution of racial and ethnic health equity research, education, and social/administrative activities in an academic institution. We mapped the network of health research scholars active in racial and ethnic health equity research and practice at the University of Rochester Medical Center (URMC; including the School of Medicine and Dentistry and School of Nursing), based on a peer nomination survey.

## Materials and Methods

This analysis underwent review by the University of Rochester (UR) Research Subjects Review Board (STUDY00007271) and was deemed exempt.

*Context:* The confluence of our institution’s commitment to the development of a Learning Health System (LHS) and to our Equity and Anti-Racism Action Plan (EARAP), resulted in a high degree of coordination and collaboration supported by our Clinical and Translational Science Institute (CTSI). In February 2021, UR CTSI established an Office of Health Equity Research (OHER) to serve as a central hub for health equity research excellence, advancing health equity, promoting new research partnerships, providing pilot funding, and developing training/technical resources, as a key part of the EARAP. We identified UR faculty with experience and/or interest in racial and ethnic health equity, to develop an inventory of potential users and contributers to this office.

*Snowball Approach:* To map the distribution of expertise in racial and ethnic equity research and practice, we conducted a snowball survey (Table 1) of faculty members at the School of Medicine and Dentistry and School of Nursing of University of Rochester with experience and/or interest in promoting racial and ethnic health equity. Each respondent could nominate other investigators who also had experience and/or conducted research/educational/capacity-building activities related to racial and ethnic health equity. Furthermore, the respondents were asked to identify their areas of expertise in *research*, *education*, or *social/administrative activities* to improve racial and ethnic equity in health, based on the following classification:

**Table 1.** The areas of expertise of participants related to racial/ethnic equity

“quantitative or qualitative research about the extent, mechanism, and impact of racial and ethnic disparities on the health of individuals or populations (e.g., epidemiologic or geographical studies of health disparities, or lived experience of individuals).” extent, mechanisms, and impact of racial and ethnic disparities extent, mechanisms, and impact of different types of racism	78 (64%) 62 (79%) 16 (21%)
“conducting research on the effect of **interventions** to improve racial and ethnic health equity.” individual-level interventions to address racial and ethnic disparities Organizational, system, and policy-level interventions to address racial and ethnic disparities individual-level interventions to tackle various types of racism Organizational, system, and policy-level interventions to tackle various types of racism	58 (48%) 40 (69%) 28 (48%) 10 (17%) 10 (17%)
“**educational efforts** to improve racial and ethnic equity in health.”	66 (55%)
“**social/administrative activities** to improve racial and ethnic equity in health (e.g., awareness-raising, advocacy, capacity-building, system reform, policy development, or evaluation).” University-level activities Local community-level activities Regional-level activities State-level activities National-level activities	61 (50%) 41 (68%) 40 (66%) 21 (34%) 17 (28%) 24 (40%)

- **Research**: involving observational research, that focuses on quantitative or qualitative study of the **extent, mechanism, and impact** of racial and ethnic disparities on the health of individuals or populations (e.g., epidemiologic or geographical studies of health disparities, or lived experience of individuals), and interventional research, focusing on the effect of interventions to improve racial and ethnic health equity.
- **Education**: involvement in formal and informal training to students, staff, faculty members, and also in the communities.
- **Social/administrative activities:** activities to improve racial and ethnic equity in health (e.g., awareness-raising, advocacy, capacity-building, system reform, policy development, or evaluation).

Each respondent could choose multiple expertise categories, if relevant. The respondents could also describe their research and practice in the free text boxes provided for each expertise category (Appendix 1).

We calculated the frequency of various health equity expertise categories (*research*, *education*, *social/administrative activities*) identified by respondents and used Cohen’s Kappa to indicate the involvement of respondents in multiple categories, beyond chance. The analysis was carried on in STATA 15.1 program [24].

*Nomination network analysis:* We transformed the identified name lists into a nomination network, in which actors (scholars) were connected to each other by nomination ties (actor A nominated actor B). We limited the analysis to part-time or full-time faculty members at the School of Medicine and Dentistry and School of Nursing of the University of Rochester, that are parts of the University of Rochester Medical Center (URMC). We calculated structural indicators of the nomination network, including density (the proportion of all possible relations that exist), and reciprocity (the proportion of relations that are bi-directional), and centrality of network actors (the number of nominations).

Given the inherent dependence of social relations in a network, traditional general linear model techniques are not suitable, and there is a need to adjust the variance of outcomes based on the dependence in the network. We used permutation techniques that involve random shuffling of the rows and columns of the matrices several times to develop a distribution of all possible network compositions under null hypothesis [25]. We used linear regression (with 10,000 random permutations) to predict actors’ centrality by their expertise categories. We applied quadratic assignment procedure (QAP) logistic regression (with 10,000 random permutations) [26] to predict nominations between pairs of actors based on the similarity of expertise categories, as well as being affiliated with the same departments.

The quantitative nomination network analysis was conducted using version 6.7 of Ucinet program for Windows [27]. We thematically analyzed the answers to the open-ended questions regarding respondents’ involvement in various expertise categories, using a descriptive qualitative approach [28]. We used the classification of expertise fields mentioned above (research, education, and social/administrative activities) as the initial coding framework. Within each category, we thematically analyzed the subject matter topics using an inductive process. The qualitative network analysis was conducted using Dedoose software (Manhattan Beach, CA) [29].

## Results

*Characteristics of participants:* In the first round of the survey, invitations were sent to 15 experts who were known by the research team as investigators involved in health equity research and practice. We received 10 responses, nominating 46 other investigators. In the second round, we sent the survey to 51 individuals, including the recently nominated investigators, as well as five of the initial contacts who did not respond to the first invitation. We received 35 additional responses, nominating 33 new investigators. Subsequently, in the third, fourth, and fifth rounds, we contacted 52, 56, and 38 individuals, respectively, who nominated 33, 30, and 14 new investigators. In the sixth and final round of the survey, we contacted 18 individuals and received 9 responses, nominating 3 new investigators. We stopped the survey after the sixth round due to information redundancy, with a total of 121 respondents.

The respondents were affiliated with 20 different departments across three schools of Medicine, Dentistry, and Nursing. The most popular among the respondents were Pediatrics (16%), Public Health Sciences (14%), department of Medicine (10%), Psychiatry (10%), Obstetrics and Gynecology (10%), and the School of Nursing (9%).

*Areas of expertise:* Of the respondents, 64% were involved in *research* related to the extent, mechanism, and impact of racial/ethnic disparities, 48% were involved in interventional *research*, 55% were involved in *education*, and 50% were involved in *social/administrative activities* (Table 1). Among respondents who were involved in *research*, a larger percentage focused on racial and ethnic disparities than studying various types of racism (79% versus 21%). *Social/administrative activities* were also more likely to happen at university and local community levels.

The overlap between expertise categories that were identified by respondents was small (Table 2). The statistically significant and positive overlap between pairs of expertise categories was observed between *education* with *social/administrative activities* (kappa: 0.27; *p* < 0.001), as well as between *research* on interventions and *education* (kappa: 0.14; *p* < 0.05). It means that there was a significant and positive overlap between choosing education and social/administrative activities, as well as between education and interventional research. Although not statistically significant, the other observed positive Kappa agreement was between doing *research* on extent and outcomes and *research* on interventions (kappa: 0.12; *p* < 0.08).

**Table 2.** The overlap between pairs of health equity expertise categories

	Research on extent and outcomes	Research on interventions	Education	Social/admin activities
Research on extent and outcomes	–	41 (34%) Kappa 0.12 (*p* < 0.08)	39 (32%) Kappa –0.12 (*p* < 0.9)	35 (29%) Kappa –0.17 (*p* < 0.9)
Research on interventions	–	–	36 (30%) Kappa 0.14 (*p* < 0.05)	29 (24%) Kappa –0.3 (*p* < 0.6)
Education	–	–	–	42 (35%) Kappa 0.27 (*p* < 0.001)
Social/admin activities	–	–	–	–

**Figure 1.**
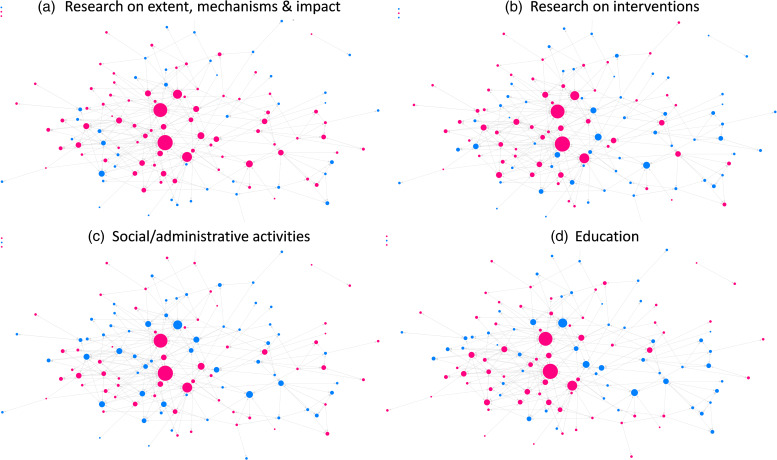
Nomination network analysis of health equity research, education, and *social/administrative* activities across the different categories. the pink nodes represent respondents who indicated involvement in research on extent and mechanisms (***a***), research on interventions (***b***), social/admin activities (***c***), and education (***d***).

*Structural indicators of the nomination network:* Visual inspection of the nomination network map of health equity investigators (Fig. 1) showed a well-connected network with a few actors who were visually more central and had no noticeable clusters. The most central actors (larger nodes) were involved in multiple expertise categories, as represented by the pink color in Fig. 1a–d. The nomination network had a density of 2% and a reciprocity of 15%.

*Predictors of nomination:* Findings from the QAP regression analysis to predict nomination ties based on shared expertise categories and shared departments among pairs of actors are provided in Table 3. Respondents were more likely to nominate someone if both were involved in research (OR: 3.1) and education (OR: 1.7) and both were affiliated with the same departments (OR: 3.7).

**Table 3.** The quadratic assignment procedure (QAP) regression to predict the likelihood of nominations based on shared expertise categories and shared department affiliations

	Coefficient ± SD (Odds Ratio)
Both involved in research	1.14 ± 0.19*** (3.12)
Both involved in education	0.50 ± 0.13*** (1.65)
Both involved in social/admin activities	0.09 ± 0.13 (1.1)
Both affiliated with the same department	1.32 ± 0.24*** (3.74)
Intercept	−5.24 ± 0.51*** (0.005)
Likelihood ratio: -1395***	

***: *p* < 0.001.

The regression analysis to predict actors’ centrality by their expertise categories showed that being involved in health equity research was the only significant predictor of actors’ centrality, with a coefficient of 2.1 (SD: 0.59, *p* < 0.001) (Table 4).

**Table 4.** Regressing actors’ centrality by their expertise categories

	Coefficient ± SD
Research	2.1 ± 0.59***
Education	0.62 ± 0.50
Social/admin activities	0.05 ± 0.50
intercept	0.48 ± 0.65

***: *p* < 0.001.

**Figure 2.**
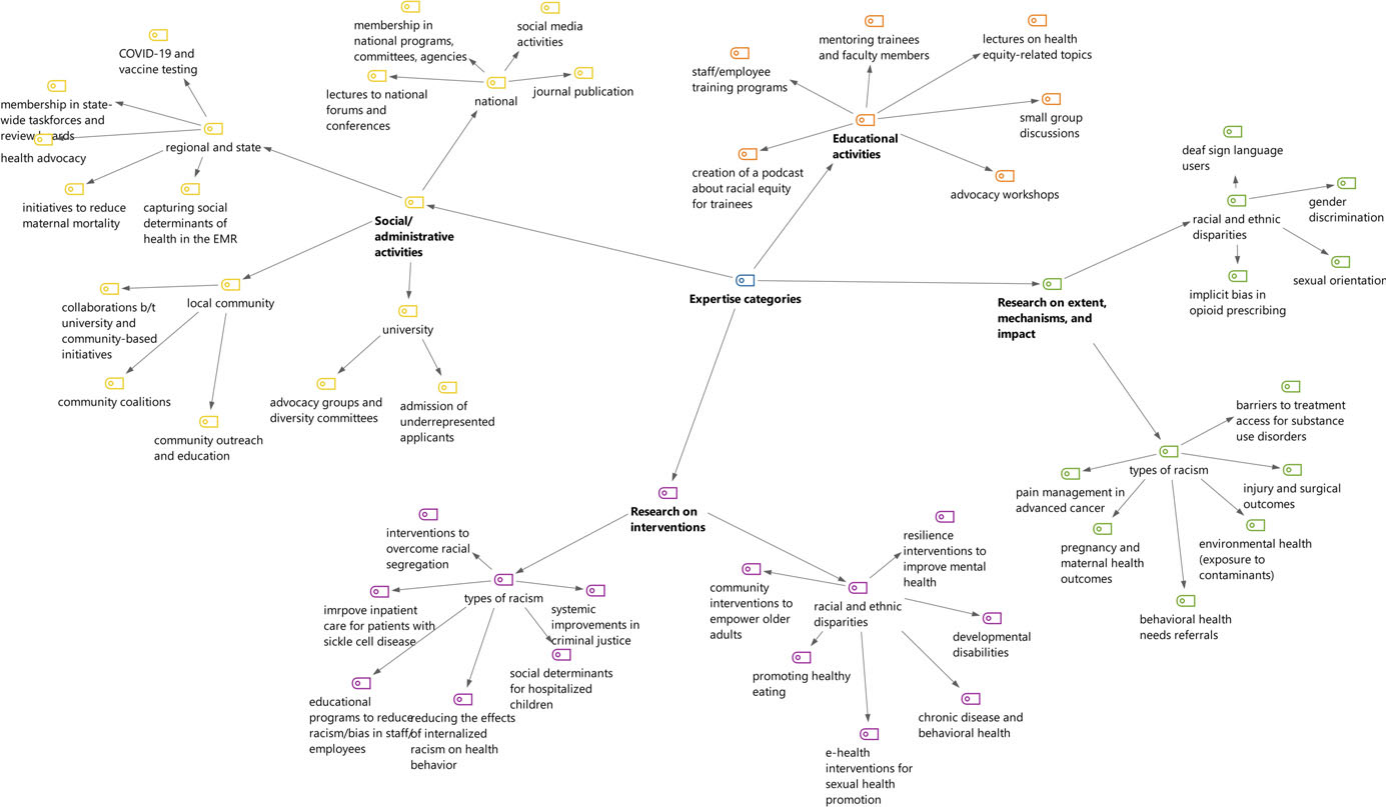
Thematic analysis of research, education, and social/administrative activities related to racial and ethnic health equity.

*Thematic content analysis:* We classified and aggregated the free text answers regarding the respondents’ areas of expertise and presented the classification in Fig. 2 and Appendix 2.

The main themes related to the *research* on the extent, mechanisms, and impact of disparities included studying access (e.g., access to COVID-19 testing or HIV treatments), disparities in health outcomes (e.g., surgical or maternal health outcomes), systemic disparities (e.g., in referral to behavioral health services), and environmental disparities (e.g., exposure to contaminants). Themes related to the extent, mechanisms, and impact of racism included the experience of racism based on gender identity and sexual orientation, the experience of Sign Language users, and racism in the context of opioid prescription.

Some themes of interventions to address disparities included strategies to improve access (to health literacy tools or preventive services), training and education, resilience building, use of e-health, and community-based activities. Interventions to tackle racism included training to reduce internalized racism among healthcare providers, systemic interventions related to criminal justice and access to legal services, and early childhood interventions to tackle racial segregation.

Themes related to *education* included course development (on advocacy, ethics, health disparities, and social justice), small group discussions, workshops, lectures, podcasts, and mentoring of medical trainees, healthcare providers, and health researchers.

We classified *social/administrative activities* based on the scope. At the university level, themes included participating in advocacy groups and diversity committees and improving inequities in students’ admission process. In the local community level, themes included community outreach and partnership. In the regional/state level, *activities* included participation in regional committees and taskforces, giving lectures at different agencies, collaboration in state-wide health promotion activities, and restructuring electronic medical records to capture social determinants of health.

In the national level, themes included participating and organizing national forums, committees, and conferences, conducting antiracism training, social media activities, and scientific publications.

## Discussion

This study mapped the network of scholars active in racial and ethnic health equity research and practice at an academic institution. We found that central actors in the nomination network were involved in multiple expertise categories. Doing *research* was the only significant predictor of popularity (i.e., being nominated by several peers). Scholars were more likely to identify others based on homophily (i.e., being affiliated with similar departments and having similar involvement in equity research and education). Respondents were more involved in *research* about racial and ethnic health equity and antiracism, than participating in local, regional, or national social/administrative activities; and being involved in *social/administrative activities* did not significantly associate with popularity. We also provided a thematic analysis of the *research, education, and social/administrative activities*, which could be used as a preliminary frame for the classification of health equity research and practice [30,31].

To advance racial equity and antiracism in academia, *research, education, and social/administrative activities* should go hand in hand. *Education* is a critical pillar in advancing equity. Academics lack an optimal recognition and understanding of racism and have an under-developed vocabulary to communicate about it [31,32]. In addition, academics usually under-recognize [33] the importance of race and racism and address them as external to their institution [5]. In our study, two groups of respondents who were involved in interventional research and in *social/administrative activities* had little overlap, while individuals who were involved in education had significant overlap with both. This implies that education can play a bridging role in connecting these less-connected specialty clusters. Our thematic analysis showed that educational activities are diverse, ranging from academically oriented education (e.g., lectures and mentorship) to community-focused (e.g., advocacy workshops and podcasts), and could serve different audience groups. Further involvement of scholars involved in research and social/administrative activities in equity education will provide opportunities for collaboration across disciplines and will improve the relevance and suitability of educational activities for different audience groups.

Racial equity and antiracism in academia require direct engagement of researchers in activities to raise awareness, build capacities, motivate and involve the leadership, and build trust and dialog with communities. However, academics tend to reflect more than they act [34]. *Social and administrative activities* are inseparable from *research* and *education*, as the engagement of community partners and minoritized researchers are critical to guide the direction of research and educational activities [31]. In our study, we found that even though individuals involved in *social/administrative activities* significantly overlapped with the ones involved in *education*, there was little overlap between *social/administrative activities* and *research*. In addition, despite other categories, co-involvement in *social/administrative activities* was not associated with peer nomination. This implies that scholars who are involved in *social/administrative activities* are dispersed and disconnected, compared to the ones who are involved in research. This could be because of the diverse nature of such *activities*, including membership in professional boards, involvement in systemic reforms, and collaboration with community-based organizations and outreach activities, as reflected in the thematic analysis. On the other hand, we found that involvement in *social/administrative activities* did not contribute to centrality, which implies that these activities are not usually well recognized and celebrated by the institution. *Social/administrative* a*ctivities* to promote racial equity and antiracism are less likely to result in publication and are often led by racially minoritized faculty members, which deepens the equity gaps in faculty retention and promotion [35,36]. Recent attention to this equity gap has resulted in movements by various academic institutions to recognize such activities in the tenure and promotion mechanisms [37,38], including at the University of Rochester [39]. In addition to these efforts, it seems that there is room for better translational synergy between the research and social/administrative activities, bridging different domains of translational spectrum [12].

We found a few investigators who were nominated the most by their peers. These central actors were involved in multiple areas, including *research, education, and social/administrative activities*. By involvement in multiple expertise categories, these central actors bridged expertise clusters. Their popularity and bridging role are two important characteristics of effective organizational change champions [40]. Network analysis was a useful tool to identify potential champions who could be engaged and motivated [41] to lead the institutional movement towards antiracism and racial equity.

There were a few limitations to this study. We limited the analysis to faculty members who self-identified racial equity as their fields of expertise and practice and nominated others with similar expertise. This method might have missed individuals who were not connected to the respondents, perhaps due to belonging to distinct professional clusters. In addition to nomination networks, future studies may also focus on collaboration networks among researchers using grant submission and coauthorship data. The indicators of centrality in our analysis are based on nomination by other experts, through a snowball process. Other central actors might have been identified if we surveyed all faculty members regardless of their expertise and involvement in racial equity research and practice. We limited the study population, for practical reasons, to the health-related faculty members, hence under-represented basic scientists, and nonfaculty investigators and educators who are also important part of the community at URMC. Finally, this study presents the patterns of nomination in one institution, which may have little generalizability.

The findings of our study could inform institutional activities to promote collaboration among faculty members about racial and ethnic health equity research and education efforts. The findings highlighted the diversity of *education* and *social/administrative activities* and their current disconnect from *research* in this academic institution. Our thematic analysis of fields of expertise can also serve as a preliminary conceptual framework for racial equity research and practice at academic institutions.

We suggest the following institutional activities as potential interventions informed by these findings:

- health investigators involved in observational and interventional research on health equity should be further invited and engaged in educational programs. This could happen through guest lecturing to present the research findings to the broader audience and also development of research excerpts to be reached to and used by broader communities.
- The findings of this nomination analysis have already informed the development of a repository of investigators involved in health equity research. These repositories will facilitate network building and peer recognition, and future collaboration.
- Involvement of researchers in education and social/administrative activities should be further recognized and celebrated by the academic institutions and be reflected in faculty promotion and evaluation frameworks.
- development of a taxonomy of research and practice in racial/ethnic equity is an important step towards the development of sub-fields in an emerging research area. Recognition of professional clusters will also inform future funding mechanisms and priority setting for health equity research and practice. Our findings can contribute to the development of a classification system.

## Supplementary material

For supplementary material accompanying this paper visit https://doi.org/10.1017/cts.2023.555. click here to view supplementary material
