# Supplementary material for: Mapping the distribution of health equity research and practice across a university: a network analysis
Source: J Clin Transl Sci. 2023 May 22;7(1):e142. doi: 10.1017/cts.2023.555 (PMC10308423; doi:10.1017/cts.2023.555)
Supplement: Supplementary file 1 [file S2059866123005551sup001.docx]

**Appendix 1.** Health equity mapping survey

| You have been identified as an individual who is researching and/or involved in activities to improve racial/ethnic equity in health. “Health equity means that everyone has a fair and just opportunity to be as healthy as possible.” (RWJF) While our focus is on race and ethnicity, we include intersectoral research that examines race and ethnicity related to intersecting disadvantage, such as socioeconomic statue, gender, geography, and national origin to name a few.  This short survey aims to identify other investigators at the University of Rochester/University of Rochester Medical Center who are currently engaged and/or experienced in **research, education and social & administrative activities to improve racial and ethnic equity in health** by using a snowball sampling approach.  It will take about 5 minutes to complete!  Health equity is a broad and diverse field and many investigators may work in silos given their subject-matter differences. The findings of this survey will be used to map the distribution of expertise in equity activities across University of Rochester, to adapt and tailor CTSI training and capacity-building services.  Please help us by identifying relevant individuals even if you think we already know them. Please list names even if you are not in contact or do not know them in person. |
| --- |
| Your first name: |
| Your last name: |
| School/Department/Center: |
| How do you assess your level of familiarity with/expertise in **research, education and practice to improve racial and ethnic equity in health**? **[please mark all items that apply]** |
| ☐ I am not conducting research and/or involved in activities to improve racial and ethnic equity in health.  *If you are not conducting research, education, or service related to racial and ethnic equity in health, feel free to skip to the last section, and suggest other individuals who are involved in this work.* |
| ☐ **Research on impact of disparities:** I am conducting quantitative or qualitative research on the extent and impact of racial and ethnic disparities on health of individuals or populations either as a primary focus or planned secondary analyses (e.g. epidemiologic or geographical studies of health disparities, or lived experience of individuals).   - The extent, mechanisms, and impact of racial and ethnic disparities ☐ - The extent, mechanism, and impact of different types of racism ☐   Please briefly describe: ………………… |
| ☐ **Interventions to address disparities:** I am conducting research on the effect of interventions to improve racial and ethnic health equity.   - Individual level interventions to address racial and ethnic disparities ☐ - Organizational, system, and policy-level interventions to address racial and ethnic disparities ☐ - Individual level interventions to tackle various types of racism ☐ - Organizational or system, and policy-level interventions to tackle various types of racism ☐   Please briefly describe: ………………… |
| ☐**Education:** I am involved in educational activities to improve racial and ethnic equity in health  Please briefly describe: ………………… |
| ☐ **Activities:** I am involved in actions to improve racial and ethnic equity in health (check all that apply; examples may include awareness-raising, advocacy, capacity-building, system reform, policy development, or evaluation).   - University level ☐ - Local community level ☐ - Regional level ☐ - State level ☐ - National level ☐   Please briefly describe: ………………… |
| Please write the names and affiliations of other individuals at University of Rochester who you consider as experienced and/or active in research or practice to improve racial and ethnic equity in health. |
| First and last name |
| First and last name |
| First and last name |
| First and last name |
| First and last name |
| Add more? Yes No |
| First and last name |
| First and last name |
| First and last name |
| First and last name |
| First and last name |

**Appendix 2.** Themes of research, education, and social/administrative activities

- Research on the extent, mechanisms, and impact of racial and ethnic disparities on the health
  - Extent, mechanisms, and impact of racial and ethnic disparities
    - Covid-19 (testing locations and access by race)
    - access to HIV treatment
    - disparities in injury and surgical outcomes
    - barriers to access treatment for substance use disorders
    - pain management in advanced cancer
    - clinical decision-making (disparities in referral for behavioral health needs)
    - environmental health (exposure to contaminants)
    - pregnancy and maternal health outcomes
  - Extent, mechanisms, and impact of different types of racism
    - implicit bias in opioid prescribing
    - deaf sign language users
    - lived experience and outcomes of racism based on sexual orientation
    - gender discrimination
- Research on the effect of interventions to improve racial and ethnic health equity
  - Interventions to address racial and ethnic disparities
    - injury prevention in underserved communities
    - better access to preventive services (e.g.HPV vaccination, HIV testing and education)
    - improving frailty in cancer
    - improve access to health literacy tools
    - community based interventions to address chronic diseases (e.g. obesity, asthma, hypertension) and behavioral health (smoking cessation)
    - E-health interventions for sexual health promotion
    - resilience-building interventions to improve mental health
    - community interventions to empower older adults
    - developmental disabilities
    - promoting healthy eating
  - Interventions to tackle various types of racism
    - systemic improvements in criminal justice
    - reducing the effects of internalized racism on health behavior
    - improve in-patient care for children and adults with Sickle cell disease
    - racial segregation, and childhood and educational interventions to overcome it (early literacy)
    - community interventions to address social determinants of health for hospitalized children
    - educational programs to reduce racism/bias in staff/employees
- Educational activities to improve racial and ethnic equity in health
  - development of courses on advocacy, ethics, health disparities, and social justice for medical trainees and PhD students
  - Advocacy workshops (e.g. workshops on White privilege for medical trainees)
  - small-group discussions
  - giving lectures on health equity-related topics
  - mentoring trainees and faculty members
  - creation of a podcast about racial equity as a requirement for trainees
  - staff/employee training programs
- Activities to improve racial and ethnic equity in health
  - University
    - University-wide or department-wide advocacy groups and diversity committees
    - University admission of underrepresented applicants
    - Development of training programs in medical education at different levels
    - University lectures
  - Local community
    - Collaborations between the university and community-based initiatives
    - community coalitions
    - local community outreach and education
  - Regional and State
    - activities about covid-19 testing and vaccine in the region
    - Restructuring electronic medical records to capture social determinants of health
    - Initiatives to reduce maternal mortality in the state
    - health advocacy in the state level
    - Membership in state-wide taskforces and review boards
  - National
    - Social media activities
    - Membership in national programs, decision-making committees, and funding agencies
    - Giving lectures in national forums and conferences
    - Publication in journals
